# The Effect of Regime Oral-Hygiene Intervention on the Incidence of New White Spot Lesions in Teenagers Treated with Fixed Orthodontic Appliances

**DOI:** 10.3390/ijerph17249460

**Published:** 2020-12-17

**Authors:** Urszula Kozak, Anna Sękowska, Renata Chałas

**Affiliations:** 1 Chair and Department of Jaw Orthopaedics, Medical University of Lublin, 20-093 Lublin, Poland; annasekowska@umlub.pl; 2 Department of Oral Medicine, Medical University of Lublin, 20-093 Lublin, Poland; renata.chalas@umlub.pl

**Keywords:** demineralization, fixed orthodontic appliances, oral hygiene, white spot lesions

## Abstract

The present study aimed to evaluate the effect of the oral-hygiene regime on the incidence of enamel demineralization in young patients treated with fixed appliances. Research was conducted in a group of 144 patients aged 12–18 years, divided into 2 groups: orthodontically treated and control. The study was divided into three stages: before treatment (I), at 1 month (II), and at 6 months (III) for their follow-up. The International Caries Detection and Assessment System (ICDAS) was used for the visual assessment of white spot lesions (WSL). After 1 month, no new white spot lesions were observed. After 6 months of the treatment, new lesions were observed in 5% of the orthodontically treated patients and in 6% of the patients in the control group. New decalcifications were located on the proximal surfaces of the central incisors, first premolars, and first molars in the orthodontically treated group; and on the lateral incisors, first premolars, and first molars in the control group. We also observed new enamel demineralization on the vestibular surfaces of the canine and first premolar in the study group. The placement of a fixed appliance did not significantly affect teeth with the presence of new white spot lesions compared to the control group during 6 months of observation.

## 1. Introduction

Orthodontic therapy carries the risk of complications. The literature shows that dental caries are the most common complication found by orthodontists. Fixed appliances may additionally predispose, initiate, or intensify this process because they limit the flow of saliva (which naturally cleanses the teeth), and provide retention places for food remains and debris [1]. Excessive material around orthodontic brackets also promotes bacterial growth. Stainless steel is characterized by high-surface tension, which can promote the development and retention of plaque deposits on the surface of the brackets. After the insertion of an appliance, changes in dental plaque are observed: an increase in the amount of carbohydrates, and in the amount of *Streptococcus mutans* and *Lactobacillus* bacteria, and a lower resting pH of biofilm.

Difficulties in removing dental plaque from the bracket area and increased accumulation of biofilm increase the risk of demineralization and decay development on the labial smooth surface [2], i.e., in the area where tooth decay does not usually develop in orthodontically untreated patients. Insufficient oral hygiene leads to the creation of a metabolically active biofilm, which disturbs the balance of the demineralization and remineralization processes, leading to the formation of white spot lesions on the enamel. An early enamel lesion (white spot lesion, white opaque spot, initial, incipient lesions) is a reversible form of tooth decay. It has the appearance of a white or brown stain on the surface of teeth. It is characterized by subsurface demineralization. Changes in light dispersion through the demineralized and porous enamel cause its white appearance [1,3]. Differences in the refraction index (RI) of enamel (1.62), water (1.33), and air (1.0) are noticeable in a visual examination. Thus, it is possible to clinically assess the degree of demineralization. A lesser degree of advancement of the change is evidenced by its visualization only after drying, but a greater degree without drying.

Characteristic changes in people wearing brackets were described as early as the 1970 s, when Zachrisson B.U. and Zachrisson S. examined a group of patients undergoing therapy with fixed appliances to determine the relationship between caries intensity and oral hygiene during therapy [4,5]. Other studies showed that enamel white spot lesions are most common on the first molar, upper lateral incisors, and lower canines, and premolars. In most cases, white spot lesions are a narrow band surrounding the base of the bracket or stretching between the bracket and the gingiva edge [3]. They are regular in shape, are sharply separated from the surrounding enamel, and occur asymmetrically in places characterized by difficult access to hygienic treatments (around the brackets, at the edge of the gingiva, and in the area of loops, springs, etc.) [6,7].

The carious enamel lesion formation takes at least 6 months [8,9]. White spot lesions are formed early. It is possible for them to be experimentally visible after 4 weeks of orthodontic treatment [3,9]. In clinical conditions, according to the literature, during the first 6 months of orthodontic therapy, a rapid increase in the number of white spot lesions on the enamel surface was observed [10,11].

The widely recommended basic method of diagnostics of caries lesions, limited to enamel (its opacity), is usually visual examination [12,13] with the use of the International System for the Detection and Assessment of Caries (ICDAS) [14].

The aim of the study was to evaluate the occurrence of enamel demineralization in young patients treated with fixed appliances.

## 2. Material and Methods

### 2.1. Eligibility Criteria

Generally, healthy patients were qualified to the study group. Because, according to the literature, age is a risk factor in the development of white spot lesions during fixed-appliance treatment [15], and because teenagers have more supragingival dental plaque, a group of patients aged 12–18 with full permanent dentition were selected for the study.

Excluded from the study were patients who were diagnosed with the following:

- previous orthodontic treatment and surgical procedures;
- noncarious enamel lesions;
- interruption of the enamel continuity; and
- poor oral hygiene.

Adequately to the study group, a control group was formed.

### 2.2. Sample Characteristics

The clinical trial involved 144 patients (at 8% of the level of maximal error) aged 12–18: 94 girls and 50 boys in total. The participants were divided into 2 groups: orthodontically treated and control. The group of orthodontically treated patients included patients qualified for treatment with conventional fixed orthodontic appliances. The control group consisted of students attending schools. In each of the schools there were dental offices.

Mean age was 14.03 for the orthodontically treated group, and 13.48 for the control group.

A total of 60 persons undergoing treatment with fixed appliances (18 boys and 42 girls), and 84 control students attending schools (32 boys and 52 girls) were examined.

The majority of participants represented the urban environment (63.3% in the orthodontic group and 70.2% in the control group).

### 2.3. Data Collection

The study was divided into three stages:

- Stage I: Preliminary examination of patients qualified for orthodontic treatment, which was carried out during a visit preceding the insertion of permanent appliance.
- Stage II: 1 month after the placement of the brackets.
- Stage III: After 6 months of treatment.

The physical examination was carried out in accordance with WHO recommendations.

Measurements were carried out by the same examiner. The examiner was trained and calibrated by an experienced examiner to diagnose and differentiate between ICDAS scores 1 and 2 before the study started. Approximately 20% of the patients were re-examined to determine intraexaminer reliability, which was found to be 0.93.

The physical examination was carried out in a dental office in the light of a shadeless lamp, using a flat mirror and periodontal probe WHO-621.

The examination was performed before and after the teeth were brushed. For this study, the amount of white spot lesions (WSL) assessed after brushing was considered final.

The activity status of caries lesions was not assessed.

**Figure 1:**
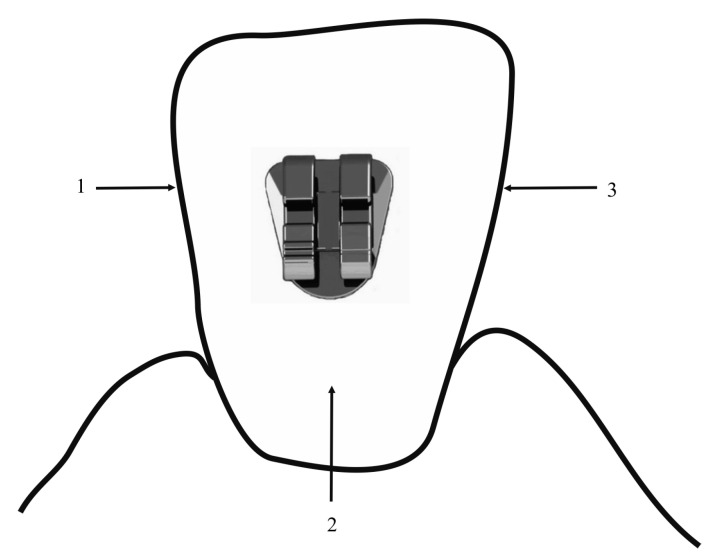
Schematic representation of examination sequence.

A new method of examining the presence of WSL in both analyzed groups was developed. The examination of patients belonging to both groups was performed in the following order: (1) observation of WSL presence was started from the mesial control surface, (2) followed by the vestibular surface, and (3) ended with the distal control tooth surface (Figure 1).

A visual assessment of the presence of white spot lesions was performed using the International Caries Detection and Assessment System (ICDAS). ICDAS 0–2 evaluation criteria were considered because patients with enamel-surface defects were excluded from the evaluation.

The codes for the detection and classification of carious lesions on smooth surfaces in accordance with the ICDAS criteria are as follows:

- Code 0: healthy tooth surface.
- Code 1: first visual change in the enamel.
- Code 2: clear visual change of the enamel when viewed wet.

As a positive result, the presence of white carious spots on enamel surfaces after drying (Code 1 according to ICDAS) and without drying (Code 2 according to ICDAS) was assumed.

Orthodontic patients were informed that optimal oral hygiene is a prerequisite for starting the therapy. Extensive oral-hygiene instruction was given prior to the start of the trials. Patients from both groups were instructed to thoroughly clean teeth with a sodium fluoride dentifrice after every meal and even after each snack, floss before brushing, and rinse once a day with a sodium fluoride mouthrinse.

The health condition of the oral cavity was evaluated in the first months of treatment in order to verify the effectiveness of the conducted hygienic procedures and show patient sites that were particularly difficult to access. Intense instructions of oral-cavity hygiene status and frequently repeated motivation was given to patients from both groups.

### 2.4. Statistical Analysis

Obtained results in the clinical trial were statistically analyzed, and are presented as descriptive analysis and U-test relations. A typical statistical-significance level of *p* = 0.05 was assumed for the entire analysis. The main calculations were performed and graphs were prepared in Statistica 8.0 PL.

## 3. Results

**Figure 2:**
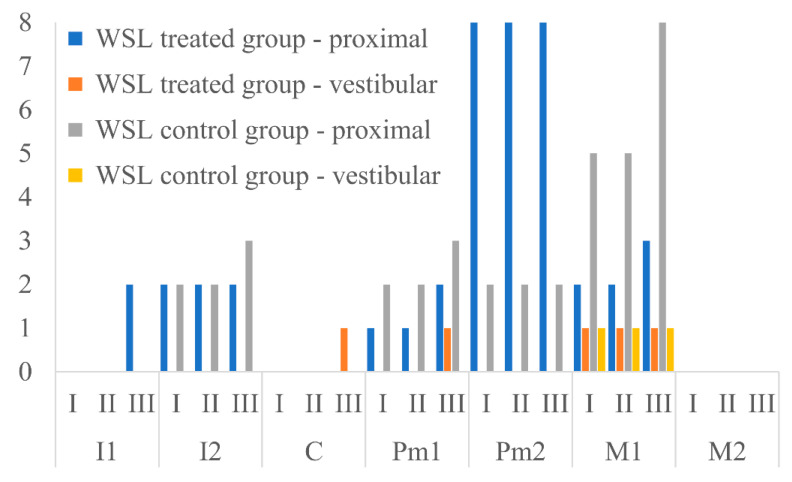
Presence of enamel decalcification (ICDAS 1–2) during 6 month follow-up in patients from both groups. I, incisors; C, canines; Pm, premolars, M, molars.

Table 1 and Figure 2 show the presence of enamel decalcification (ICDAS 1–2) in patients from both groups during 6 months of observation.

### 3.1. Anterior Teeth

After 1 month of observation, no new enamel decalcification on incisor teeth or canines was observed in patients belonging to either analyzed group. After 6 months of observation, two white spot lesions on the proximal surfaces of the central incisors and one white spot lesion on the vestibular surface of the canine appeared. All changes were observed in orthodontically treated patients. In patients belonging to the control group, one new change of demineralization character was observed. The white spot lesion was located on the proximal surface of the lateral incisor. No new enamel decalcification was observed after 6 months of observation on canines in patients belonging to the control group.

### 3.2. Posterior Teeth

After 1 month of observation, no new enamel decalcification on premolars was observed in patients belonging to either analyzed group. After 6 months of observation, three new decalcifications appeared. One of the changes was observed on the vestibular surface, and another on the proximal surface of the premolar of the first orthodontically treated patient. The third lesion, found in a control-group patient, was located in the proximal surface of the first premolar.

After 1 month of observation, no new enamel decalcification was observed on molars. However, after 6 months, 4 white spot lesions were observed. One was located on the proximal surface of the first molar in an orthodontically treated patient, while 3 more changes appeared on the proximal surface of the first molars in patients from the control group.

**Figure 3:**
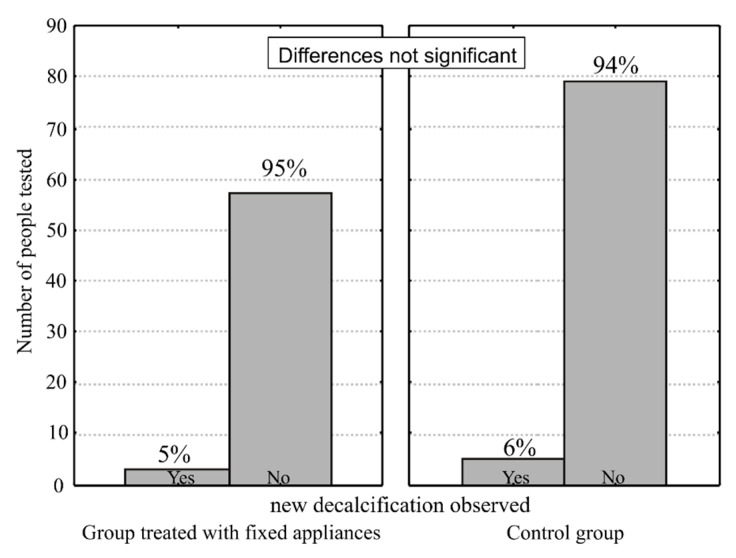
Graphical representation of people tested with new enamel decalcification.

On the basis of the U-test, we found that the percentage of white spot lesions of individual examined teeth in subsequent examinations did not significantly differ in the orthodontically treated and control groups (Figure 3).

## 4. Discussion

Good oral hygiene is an important factor affecting oral health, especially in orthodontically treated patients, because permanent appliances create additional retention spaces and make it difficult to clean teeth. Therefore, the condition of oral hygiene and the intensity of dental caries in orthodontic patients have been a topic of great importance for many years. Scientists have been discussing the influence of fixed appliances on oral hygiene and the presence of new caries defects [5,6]. The results of their study showed that treatment with fixed appliance does not significantly affect the frequency of dental caries, but changes the location of the lesions. In orthodontic patients, more frequent changes are observed on the vestibular surface, i.e., in the place where caries in patients not treated orthodontically usually do not develop. Positive correlation was also noted between the value of plaque indices, and gingiva health indices and caries indices in orthodontic patients. Although many new and improved techniques, materials, and diagnostic aids have been introduced to orthodontics in recent years, dentists still have to deal with the problem of demineralization around orthodontic brackets.

The presented results showed that the increase in the number of white spot lesions on the enamel surface in both groups did not significantly differ. However, several new demineralization centers were established after 6 months of therapy. In the study group, new decalcification appeared on the vestibular surface, near orthodontic brackets, or in the area between the brackets and the gingiva margin, which was not observed in the control group. This was confirmed by results of previous studies [4,15] indicating a change in the location of enamel decalcification in orthodontic patients. In the present study, after six months of observation, the increase in the number of white spot lesions on the surface of the enamel was not significant. Lucchese and Gherlone [9] showed that the first 6 months are of particular importance in WSL development because the majority of adolescent patients need to adapt their hygienic practices to the requirements of orthodontic therapy. Their examination revealed that there were no significant differences in WSL prevalence between patients treated for 6 and 12 months. According to studies by Tufekci et al. [10], during the first 6 months of orthodontic treatment, a rapid increase in the number of patients with at least one WSL on the enamel surface was observed. During the next 6 months, this number continues to grow, but at a slower rate. Therefore, it can be expected that the increase in enamel demineralization during the further treatment of orthodontic patients participating in the study will not be significant. The literature review indicates a large discrepancy in results obtained by various researchers. The frequency of enamel decalcification in orthodontic patients is estimated to be at 2–96%. A survey conducted by Hamdan et al. [16] showed that, in the opinion of generally practicing dentists, 20% of patients on average had white spot lesions after orthodontic treatment, whereas according to orthodontists, about 10% of patients had white spot lesions after orthodontic treatment. Enaia M. et al. [17] investigated WSL developing on the maxillary front teeth during multibracket-appliance treatment. Their examination revealed that the incidence of WSL during orthodontic therapy was 60.9% of patients. New demineralization centers in a group of patients aged 12–18 years with poor oral hygiene were observed by Fornell et al. [18]. Sagarika N. et al. [19] found a high WSL prevalence rate in 75.6% of patients undergoing orthodontic treatment. Khalaf K. [20] showed an incidence of at least one WSL in 42% patients. The study of Julien et al. [21] revealed that 23.4% of the patients developed at least one WSL during their course of treatment. Assessing the presence of white spot lesions on the labial surface of 8 front teeth, Chapman et al. [22] showed the presence of at least one demineralization focus in 36% of cases. Richter et al. [23] reported that 72.9% of patients had at least one white spot lesion during their orthodontic treatment. Hadler-Olsen et al. [24] showed the presence of at least one white spot lesion in 60% of orthodontic patients, and Al Maaitah EF et al. [25] found the presence of white spot lesions in 71.7% of patients. Such a high percentage can probably be explained by the fact that the research was carried out after the end of treatment. The high variability of results obtained by different authors can be attributed to a large variety of methods used to assess white spot lesions. Whether idiopathic enamel changes were considered is also important.

The health of the oral cavity depends on the health behavior of the individual. An evaluation of oral-cavity health condition in the first months of treatment, intense instruction of oral-cavity hygiene, and frequently repeated motivation can be seen as causes of the improvement of individual health behavior that enables to control demineralization during fixed orthodontic therapy and keeps it on a low level, similar to that of untreated patients. The presence of new WSL in some patients in both groups can be attributed to their noncompliance. This is evidenced by the fact that new WSL appeared not only on the labial, but also on the mesial surfaces, considered as control.

Most of the examined patients included in both groups were female. This may suggest the role of gender in the process of enamel demineralization. This gender difference may be due to commonly reported better oral-hygiene standards in females than those in males.

The results of a survey conducted by Hamdan et al. [16] showed that 56% of orthodontists rarely and 7% never removed brackets due to poor oral hygiene in the patient. According to the literature, white spot lesions do not disappear after brackets are removed and oral hygiene is improved. Studies by Mattousch TJ et al. [26] showed that, although in two-fifths of cases changes show some improvement, the majority of white spot lesions are irreversible, and the condition of 15% of patients even worsens after two years of retention. The extent of changes in mineralization is the highest in the first 6 months after the removal of the fixed appliance. White spot lesions visible after this time do not disappear. Orthodontic therapy is inextricably linked with normal hygienic behavior. The dentist’s task is to make the patient aware of the rules of oral hygiene in order to prevent the negative consequences of orthodontic therapy. It is important to identify patients with poor oral hygiene and to implement intensive prophylactic programs before orthodontic therapy begins. It is worth quickly reacting to the patient’s decalcification in order to prevent the progression of changes. Treatment should not be started unless there is a reasonable chance that the patient would benefit from it [27]. The presence of white spot lesions affects the perception of treatment results not only by the patient, but also the opinion of other dentists about the work of the orthodontist.

## 5. Conclusions

The present study has some limitations.

- additional analysis for maxillary and mandibular teeth was not made;
- sample size was not large enough to detect practical difference when one truly existed.

Bearing such limitations in mind, we can conclude the following:

1. In the presented model of the study, the placement of a fixed orthodontic appliance did not significantly affect teeth with the presence of new enamel-decalcification lesions compared to the control group during 6 months of observation.
2. An evaluation of oral-cavity health condition in the first months of treatment, intense instructions on oral-cavity hygiene, and frequently repeated motivation improved the individual health behavior and enabled to control demineralization during fixed orthodontic therapy.
3. In patients treated with a fixed appliance, white spot lesions most often appeared on the vestibular surface of teeth of the examined teenagers.

## Figures and Tables

**Table 1:** Patients with white spot lesions (WSL) in orthodontically treated and control groups.

Tooth	Test	Percentage of Patients with WSL		*p*
		Orthodontically Treated Group	Control Group	
I_1_	I	0.0%	0.0%	1.0000
	II	0.0%	0.0%	1.0000
	III	1.7%	0.0%	0.8437
I_2_	I	3.3%	2.4%	0.9102
	II	3.3%	2.4%	0.9102
	III	3.3%	3.6%	0.9775
C	I	0.0%	0.0%	1.0000
	II	0.0%	0.0%	1.0000
	III	1.7%	0.0%	0.8437
Pm_1_	I	1.7%	2.4%	0.9326
	II	1.7%	2.4%	0.9326
	III	5.0%	3.6%	0.8656
Pm_2_	I	6.7%	2.4%	0.6117
	II	6.7%	2.4%	0.6117
	III	6.7%	2.4%	0.6117
M_1_	I	3.3%	6.0%	0.7564
	II	3.3%	6.0%	0.7564
	III	5.0%	9.5%	0.5915
M_2_	I	0.0%	0.0%	1.0000
	II	0.0%	0.0%	1.0000
	III	0.0%	0.0%	1.0000

C = canines; I = incisors; Pm = premolars; M = molars.
